# Race trouble: experiences of Black medical specialist trainees in South Africa

**DOI:** 10.1186/s12914-016-0108-9

**Published:** 2016-12-03

**Authors:** Nicola Thackwell, Leslie Swartz, Sipho Dlamini, Lebogang Phahladira, Rudzani Muloiwa, Bonginkosi Chiliza

**Affiliations:** 1 Department of Psychology, Stellenbosch University, Private Bag X1, Stellenbosch, 7602 South Africa; 2 Faculty of Medicine, University of Cape Town, Cape Town, South Africa; 3 Department of Psychiatry, Faculty of Medicine and Health Sciences, Stellenbosch University, Cape Town, South Africa; 4 Faculty of Medicine, University of Cape Town, Cape Town, South Africa; 5 Department of Psychiatry, Faculty of Medicine and Health Sciences, Stellenbosch University, Stellenbosch, South Africa

**Keywords:** Black doctors, Transformation in higher education, Systemic racism, Medical training

## Abstract

**Background:**

This research aimed to identify and explore the experiences of Black registrars in their training in the Western Cape’s academic hospitals in order to identify structures, practices, attitudes and ideologies that may promote or impede the advancement of Black doctors into specialist medicine. This is justified by the requirement for universities to work towards monitoring and evaluating efforts to create non-discriminatory and inclusive training environments.

**Methods:**

This study employed qualitative research methods. Ten Black African medical specialists were interviewed about their training experiences in two university training hospitals in the Western Cape Province, South Africa. Interview data was collected using open-ended questions and coded and analysed using thematic and critical discursive analysis techniques.

**Results:**

Four experiential themes emerged from the interview data, they included: 1) experiences of *everyday racism* during work hours, 2) the physical and psychological effects of tokenism and an increased need to perform, 3) institutional racism as a result of inconsistent and unclear methods of promotion and clinical competence building, and 4) an organisational culture that was experienced as having a race and gender bias.

**Conclusion:**

This is a pilot study and there are limits on the generalizability of the data due to the small sample. What is clear from our participants, though, is the strong experiential component of finding it challenging to be a Black trainee in a White-dominated profession. We are undertaking further research to explore the issues raised in more detail.

## Background

Twenty years into democracy in South Africa, the medical profession, especially at higher levels, remains dominated by White men [1–3]. A strong human resource base that includes racial and gender diversity among medical practitioners is crucial if healthcare needs are to be met in diverse societies [4, 5]. This issue is especially pressing in South Africa with its history of racial oppression and the exclusion of Black^1^ people from the professions [1–3]. The retention and development of Black and/or female doctors is an important objective for a number of reasons. Race and gender concordant doctor/patient interactions may benefit patients due to better communication and improved understanding of social and cultural circumstances that may impact on treatment [6–9]. Increased trust and patient involvement may result in more accurate diagnoses, and may improve treatment adherence [10–12]. Students who train in a multicultural environment report feeling more confident with patients from different cultural groups, and race strongly influences training experiences [5, 13–17].

Substantial steps have been taken to improve racial diversity in undergraduate medical training since the end of Apartheid, but it appears that racial diversity amongst specialists, who train at the graduate level, remains a problem [1]. Concern has been raised in South African media recently over reports of substandard training for Black specialists and institutional racism in certain departments has been suggested to be behind claims that Black registrars are not being granted sufficient opportunity to gain the necessary clinical experience prior to sitting their qualifying examinations [18].

Specialist medical training is a lengthy, arduous process for all registrars (specialist medical trainees, known as residents in some parts of Europe and the USA), but Black registrars may experience the training environment as especially challenging or unwelcoming [19]. In a study of the experiences of Black residents in specialist training at a United States medical school, Liebschutz et al. [20], report that Black residents may experience both overt and covert discrimination from both colleagues and patients. Overt instances of racism may take the form of racial slurs and name calling [21]. In some instances patients may request a White physician after first being assigned to a Black resident. Black residents also report being mistaken for a non-physician such as a cleaner, or a medical professional of lower rank, particularly when female [20].

This qualitative study explored the training experiences of a group of Black African specialists who have completed registrar training in the Western Cape province of South Africa within the past five years. As a first step in a larger project on race and medical specialist training in South Africa, we were interested in learning about the experiences of Black trainees who had succeeded in the system as it currently exists.

### Theoretical framework

The theoretical framework used for this study is the ‘Race trouble’ paradigm as developed by Durrheim and colleagues [21]. Durrheim et al. [21] explain that when critically analysing discourse, especially in a racially divided and marked context, the focus of analysis lies in the contextual use and social function of the discourse, rather than in its accuracy or truth. Thus it is important to state that the purpose of this analysis is not to empirically support or refute claims of racism in Western Cape training hospitals, but rather to document experiences of what Durrheim et al. [21] term ‘race trouble’, which can be read as a text that provides clues as to how these experiences occur and what could underpin them. Thus the experiences that participants articulated are viewed and presented in relation to discourses around the medical profession as a site for the reproduction of power.

## Methods

Registrar training typically involves four to six years’ clinical training at a university hospital. All participants in this study completed their registrar ships through one of the two universities in the Western Cape province of South Africa which offer medical training. Registrars are trained through a combination of practical experience and formal lectures, under the supervision of senior consultants.

Participants were recruited using purposive selection techniques. Invitations to participate were sent out by email to Black registrars who had completed training at either of the training sites in the preceding 5 years. Ten participants responded and agreed to be interviewed. The group consisted of three females and seven males, their ages ranged from 33–42 with a median age of 35, from a range of surgical and medical departments, the details of which are not revealed here as the small numbers of eligible participants could lead to their being easily identified.

Individual interviews were conducted using open-ended questions which aimed to create a conversation whereby participants could describe their experience of registrar training in their own words. Interview recordings were transcribed verbatim and analysed in accordance with the principles of thematic and critical discourse analysis. In accordance with the principles of critical discourse analysis, attention was paid to (a) the ways in which participants positioned themselves and their narratives within discourses around the medical profession and (b), in relation to ideologies that reproduce power disparities such as race and gender.

Ethical approval for this study was obtained from the Stellenbosch University Human Research Ethics Committee (HS917/2013), particular consideration was given to protecting the privacy of this small, and easily recognisable group. The study was conducted under the auspices of the Harambee project, an initiative by Black medical specialists to promote academic excellence amongst Black medical specialists in South Africa.

## Results

We identified four main themes in the data, each of which will be presented in turn.

1. **Experiences of everyday racism during registrar training**
  All participants reported experiencing some form of race trouble in the hospitals where they trained. While there were few reports of overtly racist behaviour; more commonly participants reported experiencing subtle racism that over time cumulatively contributed to a feeling that the working environment was unwelcoming towards Black specialists. This was explained as an overall climate of subtle intolerance that was difficult to pinpoint. The perpetrators of what was experienced as racist behaviour ranged from fellow doctors, to nurses, allied staff, patients and family members. Many of the participants had the perception that others viewed them as not entirely good enough, to be training as specialists and they were regarded as incompetent affirmative action appointees regardless of their achievements and prior experience. As one participant stated : ‘I’m here on merit, and tell me where that merit isn’t in me?’As a female participant explains affirmative action was often not viewed positively:
  There was a lot of grumbling at some points. I remember a time when some White registrars would not get a post, because there had been Black applicants who were meant to be pushed to the front of the queue, so to speak. And there would be a lot of grumbling, in terms of “why it is that these people should be allocated posts sooner than I would get a post, considering that I probably am better than they are”.
  Subtle racism was also experienced through a perceived lack of respect for the Black registrars’ clinical decisions. For example, a nurse might get a second opinion from a White doctor before carrying out orders, or decisions would be made about a patient without including the assigned Black doctor.
2. **Race fatigue, tokenism and the internalisation of racism**
  Feeling pressure to prove oneself during registrar training is a common experience among all registrars who want to demonstrate their skill to their superiors. However if the work environment is perceived as hostile or intolerant to Black trainees, then it is conceivable that the pressures, and resultant stress, will be greater. The term *race fatigue* has been used to describe a sense of dissatisfaction with being the token Black face in a White-dominated workplace [22]. Representing the ‘colour factor’ can lead Black employees to feel over-extended, scrutinised and undervalued. A female specialist recalls her experience of training:
  More than anything, it made me feel exhausted because every day you are trying to prove yourself. You wake up on a daily basis with the mission to show, I qualify to be here, and it’s not because I’m Black, and I got given this post on a platter. I have experience.
  A major obstacle for Black registrars during training was the paucity of Black role models in the higher echelons of academic medicine. According to participants, the low number of registrars had a knock on effect and led to a dearth of Black academics, which perpetuated a cycle of a lack of Black mentorship:
  It becomes a game of numbers. If you don’t have enough trainees that are training from South Africa, who are Black South Africans going through these departments, then you are far less likely to end up with an academic that’s been derived from that group; it’s one Black guy out of 19 White guys in the unit.
  Since qualifying as specialists, some of members of the participant group had become consultants at training hospitals and we asked how they felt about performing a mentorship role for the next generation of Black registrars. While some said that they were gratified to be doing so, one woman articulated that the role was complicated, and she was resentful of the ‘Black role-model’ label and wished that she could just be acknowledged as, simply, a good doctor.
3. **Institutional obstacles to career development**
  Structural arrangements within registrar programmes contributed to feelings that Black trainees were not welcome, or perceived as less competent. Some registrars felt that they were discriminated against if methods of drawing up schedules for specific rotations were not transparent or seemed inconsistent. As one participant stated : ‘We waited for our turn, which sometimes never came’.
  Some felt that they did not receive the same senior privileges and clinical opportunities that seemed to be afforded to more junior White registrars. Shifting goal posts and contradictory standards from one year to the next were said to be attributed to an environment where rules could be bent seemingly in favour of promising (usually White) doctors. Because consultant physicians (who are responsible for the training of specialists) are among a powerful intellectual and sociocultural elite, they hold the power to both make and bend the rules at their professional discretion, and it may be difficult for more junior staff to call them to account for their decisions. Participants felt that this freedom could be misused to discriminate subtly against Black registrars, if clear departmental guidelines were not in place. One participant said, ‘For career progression in surgery I strongly felt that everybody else was a supporting act for the Caucasians in the group’. A female specialist shares a similar experience:
  From when we started, we (the Black registrars) would always land up with the list that is for junior people, even though you had completed your two years of being a registrar. And you would find that our Caucasian counterparts would end up with lists that are meant to be…or we’d been told are meant to be for senior people, and yet they aren’t necessarily the senior people.
  Reports of unpredictable and unfair promotion within some specialist departments emerged in many of the interviews, along with reported experiences of decreased access to research opportunities, surgical experience, and favourable clinical rotations. Senior registrar privileges, such as time off from ward duties to study for examinations, were in some instances perceived to be granted only to White registrars. Whites were sometimes seen to be fast tracked through the system despite being more junior additions to the department. One participant reported that in his surgical department White registrars were always divided across the different rotations so that they by default became the ‘stars of the show’ with the Black students performing a ‘supporting role’ in whatever procedure they performed. This anecdote demonstrates how a seemingly ‘fair’ practice of assigning groups with a racial spread may echo and reinforce racial stratification in society, where White people tend to be assigned leadership roles and Black people to do more menial work.
  The influence of leadership was viewed as playing an important part in reworking structures and routines so as to minimise institutional racism which tended to creep in when processes were implicit, unquestioned or constructed as ideologically neutral. One participant explained:
  Coming into an environment like this, I understood that you have people who are used to one particular culture, and that comes with a culturally dominant way of thinking. So I didn’t expect people to appreciate that excellence or good work would come from a different kind of place, that they don’t recognise.
4. **Male centred discourse in the culture of medicine**
  When we asked female participants how they experienced the work environment, a common reaction was the need to ‘keep your head down’, ignore it and work harder in order to ‘get on with the guys’. Participants also commonly spoke of having to ‘sink or swim’, as they put it, discursively evoking an image of male stoicism and hardiness similar to that valued in the military and other male dominated organisations. Strength, competitiveness, efficiency and emotional detachment, as well as the observance of a strict hierarchical structure, were experienced as institutional values. Toughness was reportedly rewarded. As one participant stated: ‘You don’t want to be labelled as someone with a chip’. Conversely, being viewed as weak, or in need of nurturing, was interpreted as needing ‘babysitting’. Complaining or ‘breaking rank’ was viewed as uncollegial, and meant that one was bringing one’s private life or ‘politics’ into the workplace. One woman participant explained:
  So what you find is that at meetings, possibly, or at ward rounds, or in any sort of situation, to get your point across you have to be a bit louder, or you have to be a bit more aggressive in trying to get your point across. Otherwise, there’s just this sort of tendency not to actually pay attention to anything that you might have to say. It takes a certain personality of a woman (especially a Black woman) to actually be a doctor.
  Thus the culture of medicine can be viewed as a means of cultural reproduction that has historically valued certain constructions of heroic masculinity, particularly that of the elite White male who was able to work all hours without competing social and domestic pressures.

## Discussion

Our findings are consistent with studies of resident experiences conducted in other countries as there is strong evidence to suggest that racial disparities influence the training experiences of medical interns globally [17, 20, 23–25]. It also expands upon similar investigations in South Africa [2, 19, 26–28]. The experiences of these doctors suggest that if the organisational culture of training institutions is experienced as alienating to Black professionals, simply increasing the number of Black trainees does not solve the problem of a sustainably diverse medical workforce. Retention in an environment experienced as hostile will be compromised.

Race trouble was noted as most often experienced as a generalised undercurrent of feeling unwelcome and unrecognized, and while some instances of overt racial slurs were reported, for the most part experiences of race trouble lurked under the surface, in a way that resulted in a somewhat demoralising workplace for Black registrars. Because *everyday* racism [29] is often experienced as the cumulative effect of a range of concealed and seemingly insignificant incidents, most participants thought that there was little room for recourse, or for voicing one’s grievances. This was due to the registrars’ perception that raising concerns with the department would be pointless because incidents could easily be denied or dismissed as coincidental.

Covert discrimination may take the form of seemingly inconsistent expectations and unfair treatment by senior staff, difficulty advancing in the department, a disregard for competence when it comes to making clinical decisions, and social isolation such as being excluded from social events, where networking takes place [20, 23].

Racist speech is often carefully constructed so as to protect the speaker from being viewed as prejudiced, thus overtly racist statements are avoided and more subtle criticisms are made, these often being attached to an anecdotal justification in an effort to give the prejudiced statement credibility [30, 31]. Negative attitudes towards affirmative action (whether real or perceived) reportedly led to the stigmatisation of recipients when their competence was subtly questioned [32–34]. The selection of students in accordance with what are viewed as racial quotas can be perceived negatively by other groups who are in competition for placements in registrar programmes, as has been reported in other professions [35].

While it is clear that both Black men and women experience instances of race trouble in training, Black male registrars may find it easier to align themselves with the male-centred culture that has historically come to define the identity of the specialist doctor. Thus race and gender intersect to doubly stigmatize Black female registrars, making their training experience even more challenging [36].What is ideologically valued in South African training hospitals comes predominantly from the tradition of western medicine established in Europe and North America. People may not place value in viewpoints of other cultures, or expect excellence to stem from them. This form of cultural imperialism universalises the experiences and outlook of the dominant group and renders it the norm. Those who are ‘other’ to this conception undergo what Young (p. 59) [37] identifies as a ‘paradoxical oppression’ as they are both marked out as different by stereotypes, and at the same time rendered invisible.

The instances of race trouble detailed in this study speak to ‘discursive and embodied routines of practice’ (p. 138) [21] whereby, in the post-Apartheid context, certain historically constructed racial identities are being invoked and contested. Using race trouble as a theoretical framework to make sense of these encounters, it is evident that since racist attitudes and stereotypes define the experiences of both Blacks and Whites, rather than singling out the racist and the non-racist, it is more helpful to interrogate the mechanisms (discourses, contexts, practices) that enable ‘race’ to be performed. For example, an ideologically defined territory of the White male, namely specialist medical practice, must now be shared with the Black subject who brings with him/her all the contradictions and complications of South Africa’s racialised past. In the same way, patients who have historically looked to the White male doctor as a symbol of authority may find it difficult to accommodate the new ‘face’ of medicine when presented with a Black woman.

## Conclusions

With race and gender diversity being more frequently represented among medical trainees it is clear that if a culture of non-support [38] is experienced by those who are ‘other’ to this conception of the ideal doctor, then it is likely that these men and women will struggle to find job satisfaction as medical specialists. This study is a very small one, based on reports of a small group, and we could well have other findings from a larger group and from White trainees and teachers. This reality affects the transferability and generalizability of the findings, and results should be read not necessarily as indicative of a general trend but as an analysis of a small corpus of data. The study forms a foundational pilot for a larger study in which we plan to examine the issues raised here more broadly, using a combination of qualitative and quantitative methods. Even if the findings here are outliers (and we suspect they are not), they lend support to the view that studying race issues and race trouble in the transformation of the medical workforce in South Africa is an important area for research.

## Footnotes

1 The word ‘Black’ is used to connote all those race groups that were disenfranchised by Apartheid and is thus inclusive of Black African, Coloured and Indian groups.
